# A hybrid gene selection method based on gene scoring strategy and improved particle swarm optimization

**DOI:** 10.1186/s12859-019-2773-x

**Published:** 2019-06-10

**Authors:** Fei Han, Di Tang, Yu-Wen-Tian Sun, Zhun Cheng, Jing Jiang, Qiu-Wei Li

**Affiliations:** 1 School of Computer Science and Communication Engineering, Jiangsu University, Xuefu Road, Zhenjiang, Jiangsu China; 2 Jiangsu Key Laboratory of Security Technology for Industrial Cyberspace, Zhenjiang, Jiangsu China; 3 School of Engineering, Nanjing Agricultural University, Weigang Road, Nanjing, Jiangsu China

**Keywords:** Gene selection, Gene scoring, Particle swarm optimization, Microarray data

## Abstract

**Background:**

Gene selection is one of the critical steps in the course of the classification of microarray data. Since particle swarm optimization has no complicated evolutionary operators and fewer parameters need to be adjusted, it has been used increasingly as an effective technique for gene selection. Since particle swarm optimization is apt to converge to local minima which lead to premature convergence, some particle swarm optimization based gene selection methods may select non-optimal genes with high probability. To select predictive genes with low redundancy as well as not filtering out key genes is still a challenge.

**Results:**

To obtain predictive genes with lower redundancy as well as overcome the deficiencies of traditional particle swarm optimization based gene selection methods, a hybrid gene selection method based on gene scoring strategy and improved particle swarm optimization is proposed in this paper. To select the genes highly related to out samples’ classes, a gene scoring strategy based on randomization and extreme learning machine is proposed to filter much irrelevant genes. With the third-level gene pool established by multiple filter strategy, an improved particle swarm optimization is proposed to perform gene selection. In the improved particle swarm optimization, to decrease the likelihood of the premature of the swarm the Metropolis criterion of simulated annealing algorithm is introduced to update the particles, and the half of the swarm are reinitialized when the swarm is trapped into local minima.

**Conclusions:**

Combining the gene scoring strategy with the improved particle swarm optimization, the new method could select functional gene subsets which are significantly sensitive to the samples’ classes. With the few discriminative genes selected by the proposed method, extreme learning machine and support vector machine classifiers achieve much high prediction accuracy on several public microarray data, which in turn verifies the efficiency and effectiveness of the proposed gene selection method.

## Background

One of the major applications of microarray data analysis is to perform sample classification between different disease phenotypes, for diagnostic and prognostic purposes [1]. However, for small size of samples in comparison to high dimensionality, along with experimental variations in measured gene expression levels, it is difficult to implement a particular biological classification problem as well as gain deeper understanding of the functions of particular genes [1]. Gene selection is one of the critical steps in the course of the classification of microarray data [2]. Selecting a useful gene subset not only decreases the computational complexity, but also increases the classification accuracy.

The methods for gene selection are broadly divided into three categories: filter, wrapper and embedded methods [3]. A filter method relies on general characteristics of the training data to select genes without involving any classifier for evaluation. Most filter methods consider each feature separately with ignoring feature dependencies, which may lead to worse classification performance when compared to other types of feature selection methods [4]. In addition to considering feature dependencies, wrapper methods take into account the interaction between feature subset search and model selection. However, wrapper methods have a higher risk of overfitting than filter ones and are very computationally intensive [5]. Embedded methods have the advantage that they include the interaction with the classification model, while being far less computationally intensive than wrapper methods [6].

Since it has no complicated evolutionary operators and fewer parameters need to be adjusted [7, 8], particle swarm optimization (PSO) [9, 10] has been used increasingly as an effective technique for global optimization in past decades. In recent years, PSO has been also implemented to perform gene selection. In [11], a combination of Integer-Coded GA (ICGA) and particle swarm optimization, coupled with extreme learning machine (ELM) was used to select an optimal set of genes. In [12, 13], binary PSO (BPSO) combined with filter method was applied to search optimal gene subsets. The method in [12] simplified gene selection and obtained a higher classification accuracy compared with some similar gene selection methods based on GA, while the method in [13] could determine the appropriate number of genes and obtained high classification accuracy by support vector machine. In [14], the Kmeans-PSO-ELM method used K-means method to group the initial gene pool into several clusters, and the standard PSO combined with ELM was used to perform gene selection, which could obtain a compact set of informative genes. Since traditional PSO is apt to converge to local minima which lead to premature convergence, the above PSO based gene selection method still has much room to improve.

To overcome the deficiencies of the above PSO based gene selection methods and obtain predictive genes with more interpretability, two gene selection methods based on binary PSO and gene-to-class sensitivity (GCS) information were proposed in [15, 16]. In the KMeans-GCSI-MBPSO-ELM [16], GCS information combined with K-means method was used to identify relevant genes for subsequent sample classification, and a modified BPSO coupling GCS information (GCSI) combined with ELM was used to select smallest possible gene subsets. Although the KMeans-GCSI-MBPSO-ELM could obtain predictive genes with lower redundancy and better interpretability, it might filter out a few critical genes highly related to samples’ classes in some cases and thus lead into worse classification accuracy [16]. To overcome the weakness of the KMeans-GCSI-MBPSO-ELM, the BPSO-GCSI-ELM [15] method also encoded GCS information into binary PSO to perform gene selection by initializing particles, updating the particles, modifying maximum velocity, and adopting mutation operation adaptively. Although the BPSO-GCSI-ELM method could avoid filtering out some critical genes, it may increase the computational cost because of the large initial gene pool.

To obtain predictive genes with lower redundancy as well as overcome the deficiencies of the above mentioned gene selection methods, a hybrid gene selection method based on gene scoring strategy and improved particle swarm optimization (PSO) is proposed in this paper. Firstly, with the initial gene pool obtained with double filter strategies, randomization method combined with ELM is proposed to score each gene, and the third-level gene pool for further gene selection is established. Secondly, an improved PSO aiming at improving the search ability of the swarm is proposed to perform gene selection. In the improved PSO, to decrease the probability of converging into local minima, the Metropolis criterion of simulated annealing (SA) algorithm is introduced to update the particles, and the half of the swarm are reinitialized when the swarm is trapped into local minima. With the compact and relevant gene pool obtained by multiple filter strategies, the improved PSO could select the optimal gene subsets with high probability. Finally, experimental results on six public microarray data verify the effectiveness and efficiency of the proposed hybrid gene selection method.

The remainder of this paper is organized as follows. The related preliminaries are briefly described in “Background” section. The proposed gene selection method is introduced in “Methods” section. “Results” section gives the experimental results on six public microarray data. Finally, the concluding remarks are offered in “Conclusions” section.

## Methods

### Particle swarm optimization

Particle swarm optimization (PSO) is a population-based stochastic optimization technique developed by Eberhart and Kennedy [9]. PSO works by initializing a flock of birds randomly over the searching space, where each bird is called a particle with no quality or volume. Each particle flies with a certain velocity according to its momentum and the influence of its own previous best position (*P*_*ib*_) as well as the best position of all particles (*P*_*g*_). Assume that the dimension of searching space is *D* and the total number of particles is *n*. Then the original PSO is described as follows

1 $$\begin{array}{@{}rcl@{}} v_{id}(t+1)&=v_{id}(t)+c_{1}\times Y_{1}()\times \left[p_{ibd}(t)-x_{id}(t)\right]\\&+c_{2}\times Y_{2}()\times \left[p_{gd}(t)-x_{id}(t)\right] \end{array} $$

2 $$ \begin{aligned} x_{id}(t+1)=x_{id}(t)+v_{id}(t+1),1\leq i\leq n, 1\leq d\leq D \end{aligned}  $$

where *v*_*i*_(*t*) and *x*_*i*_(*t*) denote the velocity vector and the position of the i-th particle, respectively, at the t-th iteration; *P*_*ib*_(t) and *P*_*g*_(t) denote the previous best position of the i-th particle and the best position of all particle, respectively; *c*_1_ and *c*_2_ are the positive acceleration constants; *Y*_1_() and *r*_2_() are random number between 0 and 1. In addition, it needs to place a limit on the velocity.

To improve the convergence performance of the original PSO, a modified particle swarm optimization [10] was proposed. An inertial weight was introduced in the velocity vector evolution equation described as follows:

3 $$ \begin{aligned} {} v_{id}(t+1)&=w_{t}\times v_{id}(t)+c_{1}\times Y_{1}()\times \left[p_{ibd}(t)-x_{id}(t)\right]\\ &\quad+c_{2}\times Y_{2}()\times \left[p_{gd}(t)-x_{id}(t)\right] \end{aligned}  $$

where *w* is the inertial weight. Shi & Eberhart [10] advised the linearly decreasing method to adjust the weight as follows:

4 $$\begin{array}{@{}rcl@{}} w(t)=w_{ini}-\frac{w_{ini}-w_{end}}{T_{max}}\times t \end{array} $$

where *t* is the current iteration number; *w*_*ini*_,*w*_*end*_ and *T*_*max*_ are the initial inertial weight, the final inertial weight and the maximum number of iteration, respectively.

### Extreme learning machine

In [17], a learning algorithm for single-hidden layer feedforward neural networks (SLFN) called extreme learning machine (ELM) was proposed to solve the problem caused by gradient-based learning algorithms. ELM randomly chooses the input weights and hidden biases, and analytically determines the output weights of SLFN. ELM has much better generalization performance with much faster learning speed than gradient-based algorithms [18, 19].

For *N* arbitrary distinct samples (*XX*_*i*_,*T*_*i*_)(*i*=1,2,…,*N*.), where *XX*_*i*_=[*xx*_*i*1_,*xx*_*i*2_,…,*xx*_*in*_]∈*R*_*n*_, *T*_*i*_=[ *t*_*i*1_, *t*_*i*2_, …, *t*_*im*_] ∈ *R*_*m*_. A SLFN with *N*_*H*_ hidden neurons and activation function *g*() can approximate these *N* samples with zero error. This means that

5 $$\begin{array}{@{}rcl@{}} {Hw}_{o}=T \end{array} $$

where

$$ \begin{aligned} & H\left({wh}_{1},...,{wh}_{N_{H}},b_{1},...,b_{N_{H}},{XX}_{1},...,{XX}_{N}\right)\\ &=\left[ \begin{array}{ccc} g\left({wh}_{1}\cdot {XX}_{1}+{b}_{1}\right) & \cdots & g\left({wh}_{N_{H}}\cdot {XX}_{1}+{b}_{N_{H}}\right)\\ \vdots & \ddots & \vdots \\ g\left({wh}_{1}\cdot {XX}_{N}+{b}_{1}\right) & \cdots & g\left({wh}_{N_{H}}\cdot {XX}_{N}+{b}_{N_{H}}\right)\\ \end{array} \right]\\ \end{aligned} $$

$$ \begin{aligned} w_{o}= \left[ \begin{array}{c} {w_{o1}}^{T} \\ \vdots \\ {w_{o_{N_{H}}}}^{T}\\ \end{array} \right] \end{aligned} \quad\quad \texttt{and} \quad\quad \begin{aligned} T= \left[ \begin{array}{c} {t_{1}}^{T} \\ \vdots \\ {t_{N}}^{T} \\ \end{array} \right] \end{aligned} \texttt{.} $$

The *wh*_*i*_=[*wh*_*i*1_,*wh*_*i*2_,...,*wh*_*in*_]^*T*^ is the input weight vector connecting the *i*-th hidden neuron and the input neurons, the *wo*_*i*_=[*wo*_*i*1_,*wo*_*i*2_,...,*wo*_*im*_]^*T*^ is the output weight vector connecting the *i*-th hidden neuron and the output neurons, and the *b*_*i*_ is the bias of the *i*-th hidden neuron.

In the course of learning, first, the input weights and the hidden biases are arbitrarily chosen and need not be adjusted at all. Second, the smallest norm least-squares solution of the Eq. 5 is obtained as follows:

6 $$\begin{array}{@{}rcl@{}} w_{o}=H^{+}T \end{array} $$

where *H*^+^ is the Moore-Penrose (MP) generalized inverse of matrix *H*.

It was concluded that the ELM has the minimum train-ing error and smallest norm of weights [18, 19]. The smallest norm of weights tends to have the best generalization performance [18, 19]. Since the solution is obtained by an analytical method and all the parameters of SLFN need not be adjusted, ELM converges much faster than gradient-based algorithm.

### The proposed gene selection method

Gene selection generally consists of two steps, which are to identify relevant genes and to tend to select smallest subsets from the relevant genes. Different from the KMeans-GCSI-MBPSO-ELM [16] and BPSO-GCSI-ELM [15] methods, a scoring criterion following the double filter strategy is proposed to select highly relevant genes in this paper, which may decrease the size of the gene pool dramatically. For selecting compact gene subset from the refined gene pool, an improved PSO with the new strategies for reinitializing the swarm and updating of the *P*_*g*_ is proposed.

**Fig. 1:**
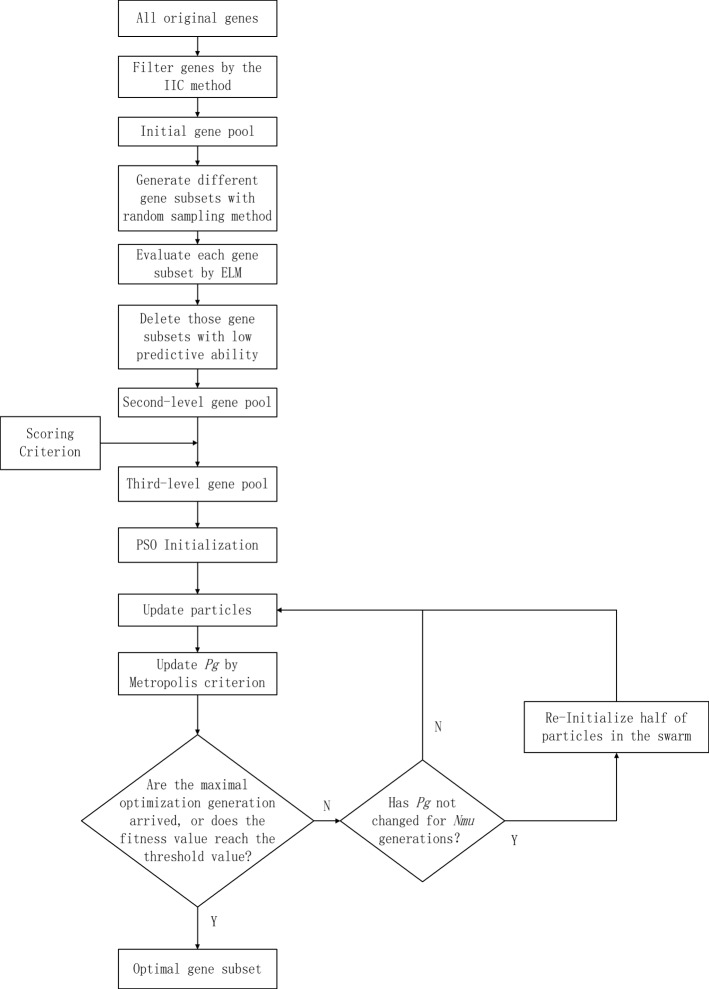
The frame of the proposed hybrid gene selection method

Since the proposed method combines the scoring criterion with the improved PSO, coupled with ELM, to perform gene selection, it is referred to as the SC-IPSO-ELM method. The rough frame of the proposed method is shown in Fig. 1, and the detailed steps are described as follows.

Step 1: Form a first-level initial gene pool. The dataset is divided into training and testing datasets. Select 200–400 genes from all original genes by using information index to classification (IIC) method [16, 20] as follows:

7 $$\begin{array}{@{}rcl@{}} d(g)\,=\,\sum_{j=1}^{c}\sum_{k=1,k=j}^{c}\left[\frac{1}{2}\frac{|\mu_{g^{j}}-\mu_{g^{k}}|}{\sigma_{g^{j}}+\sigma_{g^{k}}}\,+\,\frac{1}{2}ln\left(\frac{\sigma_{g^{j}}^{2}\,+\,\sigma_{g^{k}}^{2}}{2\sigma_{g^{j}}\sigma_{g^{k}}}\right)\right] \end{array} $$

where $\phantom {\dot {i}\!}\mu _{g^{j}}$ and $\phantom {\dot {i}\!}\mu _{g^{k}}$ are the means of expression value of the gene *g* in the *j*-th and *k*-th classes, respectively, and $\phantom {\dot {i}\!}\sigma _{g^{j}}$ and $\phantom {\dot {i}\!}\sigma _{g^{k}}$ are the standard deviations of expression value of gene *g* in the *j*-th and *k*-th classes, respectively. *c* is the total number of classes. From [16, 20], the higher the value of *d*(*g*), the more classification information the gene *g* contains, so the gene *g* is more relevant to samples categories. The high classification accuracy will be obtained with high probability by a classifier if the microarray data is projected onto the gene *g* whose IIC value, *d*(*g*), is high. The genes are ranked by their IIC values on the training dataset, and those genes with higher IIC values are chosen to establish the first-level gene pool.

Step 2: Establish a second-level initial gene pool. Randomly generate different gene subsets from the first-level gene pool. Then, each gene subset’s predictive ability is evaluated according to the 5-fold cross validation (CV) classification accuracy obtained by ELM on the training dataset projected onto the gene subset. When the 5-fold cross validation classification accuracy is less than the predetermined value (*θ*_*ac*_), the corresponding gene subset is deleted. Thus, the genes in the remained gene subsets have comparatively high predictive ability and form the second-level initial gene pool. The number of the gene subsets in the second-level gene pool is noted as *l*_*se*_. Each gene subset is ranked as integer number (from 1 to *l*_*se*_) according to the corresponding 5-fold cross validation classification accuracy. The higher the classification accuracy is, the smaller the rank number of the corresponding gene subset is.

Step 3: Establish a third-level initial gene pool by scoring strategy. The psedo-code of the scoring rule for the i-th gene in the second-level gene pool is listed as Algorithm 1.


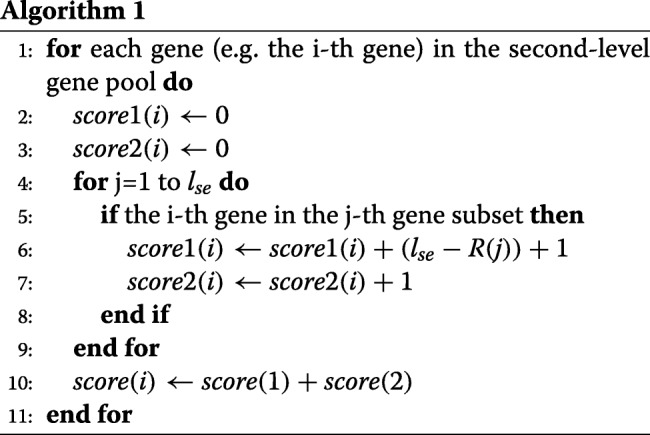
 where *R*_*j*_ is the ranked number of the *j*-th gene sub-set in the second-level gene pool. After obtaining the scores of all genes in the second-level gene pool, they are normalized into the interval of [0, 1] with linear transformation. Obviously, the higher value of the gene score is, the more relevant to the samples classes of the gene is. Further filter out those genes with much lower score values, and the remaining genes in the second-level pool form the third-level gene pool.

Step 4: Use an improved PSO to select the optimal gene subsets from the third-level initial gene pool. The i-th particle *X*_*i*_=(*x*_*i*1_,*x*_*i*2_,…,*x*_*iD*_) represents a candidate gene subset, and the element *x*_*ij*_ is the serial number of the selected gene. The dimension of the particles is equal to the number of the selected genes from the third-level initial gene pool, which is predetermined according to [15, 16]. The fitness function of the i-th particle, *f*(*X*_*i*_), is the 5-fold cross validation classification accuracy obtained by ELM on the training dataset projected onto the selected gene subset represented by the i-th particle. The optimization process of the improved PSO is the same as the traditional PSO except the following respects.

One is the strategy of updating the best position of the swarm. To decrease the probability of premature convergence of the swarm, the Metropolis criterion in SA [21] is introduced to update the best position of the swarm. In the (i+1)-th optimization generation, the best position of the swarm, *p*_*g*_, is updated by the Eq. 8 as follows:

8 $$ {\begin{aligned} p_{g}(i+1)=\left\{ \begin{array}{lr} X_{j}, & f(X_{j})-f(p_{g}(i))\geq \varepsilon\\ X_{j} \,\texttt{with the} \,P = e^{-\frac{|f(X_{j})-f(p_{g}(i))|}{T(i+1)}},& |f(X_{j})-f(p_{g}(i))|< \varepsilon \end{array} \right. \end{aligned}}  $$

where *T*(*i*+1) is the annealing temperature which decreases linearly as the following equation

9 $$\begin{array}{@{}rcl@{}} T(i+1)=T_{0}-\frac{T_{0}-T_{end}}{{It}_{max}}\times (i+1) \end{array} $$

In Eq. 7, *T*_0_, *T*_*end*_, and *It*_*max*_ are the initial annealing temperature, final annealing temperature and maximum optimization generation number.

The other is the strategy of mutating the swarm. When the swarm converges to the local minima, the particles in the swarm are close to each other, and the swarm loses its diversity. Mutating the swarm makes the particles repel each other and improves the diversity of the swarm, so the swarm jumps the local minima with high probability. In the improved PSO, the mutation operation is taken if the global best fitness value of the swarm does not change for predetermined generations (*Nmu*), which randomly select half number of particles in the swarm to reinitialize.

The SC-IPSO-ELM method firstly identifies the relevant genes by the randomization method combined with ELM. Then, with the proposed gene scoring criterion, the much more relevant and compact gene pool is obtained. Finally, to obtain the optimal gene subsets, the tradition PSO is modified to improve its global search ability. Although the SC-IPSO-ELM method does not encode prior information to perform gene selection as the KMeans-GCSI-MBPSO-ELM [16] and BPSO-GCSI-ELM [15] methods, it could also select the most predictive genes with low redundancy effectively. Moreover, the multiple filter strategies produce much more compact gene pool than the methods in [15, 16], which could decrease the computational cost of PSO searching the optimal gene subsets. Compared to the gene-to-class sensitivity information, genes’ rank information obtained by the scoring strategy is more robust, so the SC-IPSO-ELM method may not filter out predictive genes with higher probability than the methods in [15, 16].

The proposed gene selection method contains filtering irrelevant genes to establish the gene pool and using PSO to select functional gene subsets from the gene pool, and its computational complexity can be calculated as follows:

10 $$\begin{array}{@{}rcl@{}} {CC}_{SC-IPSO-ELM}=O(N_{TG}\times N_{Train})+O(l\times N_{g1}) \\ +O(l_{se}\times N_{g2})+O(N_{PSO}\times {Iter}_{PSO}) \end{array} $$

where *N*_*TG*_,*N*_*Train*_,*l*,*N*_*g*1_,*l*_*se*_,*N*_*g*2_,*N*_*PSO*_ and *Iter*_*PSO*_ are the number of the original total genes, the number of training data, the number of the initial randomly generated gene subsets in Step 2, the size the first-level gene pool, the number of the selected gene subsets in Step 2, the size of the second-level gene pool, the swarm size and the maximum iteration number in the improved PSO, respectively. The four items on the right side of Eq. 10 are the computational complexity of Step 1, Step 2, Step 3 and Step 4 of the proposed method, respectively. The first and fourth terms are as the same as those of the methods in [15, 16]. The *N*_*g*1_ and *N*_*g*2_ both are much smaller than *N*_*TG*_. Generally, the *l* and *l*_*se*_ are not greater than *N*_*Train*_. The computational complexity of the SC-IPSO-ELM method can be approximated as the sum of the first and fourth terms on the right side of Eq. 10 which is similar to the methods in [15, 16], so the time complexity of the proposed method is at the same order of magnitude of that of the methods in [15, 16]. Since the third-level gene pool is established by multiple filter strategy, the size of the third-level gene pool is small. The small third-level gene pool leads to small *N*_*PSO*_ and *Iter*_*PSO*_, which may decrease the computational cost of Step 4.

## Results

### Datasets

To verify the effectiveness and efficiency of the proposed gene selection method, we conduct experiments on the six public microarray datasets including Leukemia, Colon, SRBCT, Brain cancer data,Lung and Lymphoma data. The detailed description of the datasets is listed in Table 1.

**Table 1:** Six microarray datasets

Data	Total Samples	Training samples	Testing samples	Number of classes	Number of genes
Leukemia	72	38	34	2	7129
Brain Cancer	60	30	30	2	7129
Colon	62	40	22	2	2000
SRBCT	83	63	20	4	2308
LUNG	203	103	100	5	3312
Lymphoma	58	29	29	2	7129

The Leukemia data [22] contains total 72 samples in two classes, acute lymphoblastic leukemia (ALL) and acute myeloid leukemia (AML), which contain 47 and 25 samples, respectively. Every sample contains 7129 gene expression values. The Leukemia data are available at https://link.springer.com/article/10.1186/1471-2105-7-228#SupplementaryMaterial.

The Brain cancer data contains 60 samples in two classes, 46 patients with classic and 14 patients with desmoplastic brain cancer. The Lymphoma data includes 58 samples where 32 patients did cured and 26 patients did not cured. Each sample in the Brain cancer and Lymphoma has 7129 genes. These two data are available at http://linus.nci.nih.gov/~brb/DataArchive_New.html.

The Colon data consists of expression levels of 62 samples of which 40 samples are colon cancer samples and the remaining are normal samples. Although original expression levels for 6000 genes are measured, 4000 genes out of all the 6000 genes were removed considering the reliability of measured values in the measured expression levels. The measured expression values of 2000 genes are publicly available at http://microarray.princeton.edu/oncology/.

The entire SRBCT data [23] includes the expression data of 2308 genes. There are totally 63 training samples and 25 testing samples, five of the testing samples being not SRBCT. The 63 training samples contain 23 Ewing family of tumors (EWS), 20 rhabdomyosarcoma (RMS), 12 neuroblastoma (NB), and 8 Burkitt lymphomas (BL). The 20 testing samples contain 6 EWS, 5 RMS, 6 NB, and 3 BL. The data are available at https://link.springer.com/article/10.1186/1471-2105-7-228#SupplementaryMaterial.

The LUNG data [24, 25] contains in total 203 samples in five classes, adenocarcinomas, squamous cell lung carcinomas, pulmonary carcinoids, small-cell lung carcinomas and normal lung, which have 139, 21, 20, 6,17 samples, respectively. Each sample has 12600 genes. The genes with standard deviations smaller than 50 expression units were removed and a dataset with 203 samples and 3312 genes was obtained [24, 25]. The data is also available at https://link.springer.com/article/10.1186/1471-2105-7-228#SupplementaryMaterial.

In the experiments on all data, the swarm size is 60, the maximum iteration number is selected as 20, the acceleration constants *c*_1_ and *c*_2_ are both selected as 1.49445, and the inertial weight varies from 0.9 to 0.4. The size of the third-level gene pool is 40 on all data. The parameter *N*_*mu*_ is fixed as 3 on all data. The values of these parameters are determined by the cross-validation runs on the training datasets and according to [15, 16].

### The prediction ability of the selected gene subsets

To verify the prediction ability of the selected gene subsets obtained by the proposed method, ELM is used to perform sample classification with some gene subsets selected by the SC-IPSO-ELM method on the six datasets. Each experiment is conducted 100 times, and the mean classification accuracies are listed in Table 2.

**Table 2:** The classification accuracy obtained by elm with different gene subsets selected by the sc-ipso-elm method on the six microarray data

Data	Selected gene subsets	5-fold CV Accuracy Mean(%) ±std	Test Accuracy Mean(%) ±std
Leukemia	4050,2642,2121	100 ±0.00	100 ±0.00
	4050,2642,1882	100 ±0.00	100 ±0.00
	4050,2642,3258	100 ±0.00	100 ±0.00
	42335,2642,1843,4050	100 ±0.00	100 ±0.00
Brain cancer	1091,798,337	90.14 ±0.036	89.62 ±0.025
	3052,973,3041,3692,4796	92.00 ±0.023	91.78 ±0.046
	4628,7129,7045,4413,798	92.29 ±0.020	90.22 ±0.022
	7129,2881,3052,865,1970,2935,4871	92.78 ±0.012	91.88 ±0.019
Colon	14,1976,1325,1993,1870,1892,653,1917,187,22,1209,1060	93.63 ±0.025	97.27 ±0.013
	377,792,14,1976,765,187,251,1110,175,53,1293,1740,200	93.00 ±0.035	98.06 ±0.013
	792,1423,14,1976,1909,1110,1589,102,107,1916,175,1151	93.73 ±0.031	98.71 ±0.013
	792,14,1976,765,1909,1524,1110,175,43,53,1293,1740,251	96.86 ±0.033	99.05 ±0.011
SRBCT	742,1003,1954,430,2050,123	100 ±0.00	100 ±0.00
	545,1955,1434,509,971,255	100 ±0.00	100 ±0.00
	1003,545,1911,153,123,1489,2161	100 ±0.00	100 ±0.00
	1955,2050,545,2144,2045,123,1489	100 ±0.00	100 ±0.00
LUNG	1765,2779,2841,1474,2045,3191,2763,2817,525,1630	98.27 ±0.014	93.33 ±0.011
	525,1493,607,2763,792,580,867,368,3279,2158,1225	98.39 ±0.023	93.47 ±0.012
	1765,883,2763,792,580,867,985,3279,2988,2045,814	98.67 ±0.021	93.60 ±0.019
	1765,525,2763,2841,1474,2583,867,985,2045,814,918	98.67 ±0.019	94.01 ±0.024
Lymphoma	152,2347,2650,5679,438,1855,5863	90.60 ±0.023	85.11 ±0.020
	1855,2828,152,2437,806,530,1102	92.36 ±0.027	89.33 ±0.019
	5279,4687,4940,5449,1133,1855,4519	93.51 ±0.022	90.47 ±0.029
	152,2437,4829,2828,6441,806,2508	93.79 ±0.020	90.45 ±0.023

From Table 2, with the small gene subsets selected by the proposed approach, ELM obtains 100% 5-fold cross validation and test accuracies both on the Leukemia and SRBCT data, With the about five and thirteen genes selected by the SC-IPSO-ELM method on the Brain cancer and Colon, respectively, ELM obtains high prediction accuracies. These results indicate that the SC-IPSO-ELM method has the ability of selecting those predictive genes highly related to samples’ classes.

### Biological and functional analysis of the selected gene subsets

The experiment on each microarray data is conducted 500 times, and the top ten frequently selected genes are listed in Tables 3, 4, 5, 6, 7 and 8 for the six microarray data.

**Table 3:** The top ten frequently selected genes with the sc-ipso-elm method on the leukemia data

Gene No.	Gene Name	Description
2354	M92287	CCND3 Cyclin D3 ∗∘
6855	M31523	CF3 Transcription factor 3 (E2A immunoglobulin enhancer bind-ing factors E12/E47)
2642	U05259	MB-1 gene ∗∘*⊲*⋆∙
4050	X03934	GB DEF = T-cell antigen receptor gene T3-delta ∗⋆
1834	M23197	CD33 CD33 antigen (differenti-ation antigen) ∗∘
1882	M27891	CST3 Cystatin C (amyloid an-giopathy and cerebral hemor-rhage) ∗∘*⊲*⋆∙
4377	X62654	ME491 gene extracted from H.sapiens gene for Me491/CD63 antigen
2121	M63138	CTSD Cathepsin D (lysosomal aspartyl protease) ∗∘*⊲*⋆
2288	M84526	DF D component of comple-ment (adipsin)
6271	M33493	Tryptase-III mRNA, 3’ end

*also selected in [15]; ∘also selected in [26]; *⊲*also selected in [22]; ⋆also selected in [16]; ∙also selected in [27]

**Table 4:** The top ten frequently selected genes with the sc-ipso-elm method on the brain cancer data

Gene No.	Gene Name	Description
798	D86961	Lipoma HMGIC fusion partner-like 2
865	D87454	KIAA0265 protein
2648	M28879	Granzyme B (granzyme 2, cytotoxic T-lymphocyte-associated serine esterase 1)
2881	M57506	Chemokine (C-C motif) ligand 1
3041	M64934	Kell blood group ∗
3052	M65254	Protein phosphatase 2 (formerly 2A), regulatory subunit A (PR 65), beta isoform
3692	U03644	CBF1 interacting corepressor
4628	U50079	Histone deacetylase 1
6571	X93036	FXYD domain containing ion transport regulator 3
7129	Z97074	Rab9 effector protein with kelch motifs

*also selected in [15]

**Table 5:** The top ten frequently selected genes with the sc-ipso-elm method on the colon data

Gene No.	Gene Name	Description
14	H20709	MYOSIN LIGHT CHAIN ALKALI, SMOOTH-MUSCLE ISOFORM (HU-MAN) ∗∘*⊲*⋆
1772	H08393	COLLAGEN ALPHA 2(XI) CHAIN (Homo sapiens)
1935	X62048	H.sapiens Wee1 hu gene
286	H64489	LEUKOCYTE ANTIGEN CD37 (Homo sapiens) *⊲*⋆
792	R88740	ATP SYNTHASE COUPLING FACTOR 6MITOCHONDRIAL PRE-CURSOR (HUMAN) ∘⋆
187	T51023	HEAT SHOCK PROTEIN HSP 90-BETA (HUMAN)
1976	K03474	Human Mullerian inhibiting substance gene, complete cds *⊲*
493	R87126	MYOSIN HEAVY CHAIN, NONMUSCLE (Gallus gal-lus)
1635	M36634	Human vasoactive intestinal peptide (VIP) mRNA, com-plete cds
698	T51261	GLIA DERIVED NEXIN PRECURSOR (Mus muscu-lus)

*also selected in [28]; ∘also selected in [29]; *⊲*also selected in [15]; ⋆also selected in [16]

**Table 6:** The top ten frequently selected genes with the sc-ipso-elm method on the srbct data

Gene No.	Gene Name	Description
742	812105	Transmembrane protein ∗∘*⊲*⋆
1003	796258	Sarcoglycan, alpha (50kD dystrophin-associated glycoprotein) ∗⋆∙
255	325182	Cadherin 2, N-cadherin (neuronal) ∘*⊲*∙
123	236282	Wiskott-Aldrich syndrome (ecezema-thrombocytopenia
545	1435862	Antigen identified by monoclonal antibodies 12E7, F21 and O13 ∗⋆*⊲*∙
1319	866702	Protein tyrosine phosphatase, non-receptor type 13 (APO-1/CD95 (Fas)-associated phosphatase)
1606	624360	Proteasome (prosome, macropain) subunit, beta type, 8 (large multifunctional protease 7) *⊲*
2046	244618	ESTs
246	377461	Caveolin 1, caveolae protein, 22kD
509	207274	Human DNA for insulin-like growth factor II (IGF-2); exon 7 and additional ORF

*also selected in [23]; ∘also selected in [30]; *⊲*also selected in [15]; ⋆also selected in [16]; ∙also selected in [31]

**Table 7:** The top ten frequently selected genes with the sc-ipso-elm method on the lung data

Gene No.	Gene Name	Description
2763	185_at	Neuro-oncological ventral antigen 1
580	39333_at	Collagen, type IV, alpha 1 ∘
792	38704_at	Cadherin 2, N-cadherin (neuronal) ∗∘
2841	32696_at	Pre-B-cell leukemia transcription factor 3
2045	35276_at	Claudin 4
2657	32648_at	Delta-like homolog (Drosophila)
1765	39722_at	Nuclear receptor co-repressor 1 ∗∘
1493	38967_at	Chromosome 14 open reading frame 2
3191	39383_at	Adenylate cyclase 6
2338	1315_at	Ornithine decarboxylase antizyme 1

*also selected in [16]; ∘also selected in [15]

**Table 8:** The top ten frequently selected genes with the sc-ipso-elm method on the lymphoma data

Gene No.	Gene Name	Description
152	M97935_5_at	Signal transducer and activator of transcription 1, 91kDa
1855	L17328_at	Fasciculation and elongation protein zeta 2 (zygin II)
2437	M18185_at	Gastric inhibitory polypeptide
2347	M14091_at	Serine (or cysteine) proteinase inhibitor, clade A (alpha-1 antiproteinase, antitrypsin), member 7
2828	M37763_at	Neurotrophin 3
5279	U83843_at	Chaperonin containing TCP1, subunit 7 (eta)
806	D86968_at	Mitogen-activated protein kinase kinase kinase 4 ∗
4092	U22178_s_at	Microseminoprotein, beta-
4940	U66559_at	Anaplastic lymphoma kinase (Ki-1)
4194	U28150_at	ATP-binding cassette, sub-family D (ALD), member 2

*also selected in [16]

From Tables 3, 4, 5, 6, 7 and 8, many genes selected by the SC-IPSO-ELM method were also selected by one or more methods proposed in [15, 16, 22, 23, 26–31]. On the Leukemia data, gene U05259, a B lymphocyte antigen receptor, encodes cell surface proteins for which monoclonal antibodies have been demonstrated to be useful in distinguishing lymphoid from myeloid lineage cells [18]. Gene M63138 is the member of the peptidase C1 family involved in the pathogenesis of breast cancer and possibly Alzheimer’s disease [18]. A muscle index can be calculated based on an average intensity of 17 ESTs in the array that are homologous to smooth muscle genes which included gene H20709 in the Colon data. Although the SC-IPSO-ELM method does not encode gene-to-class sensitivity (GCS) information extracted from the microarray data, it could also select some genes with comparatively high GCS values selected by the GCSI-based methods. Since the expression levels of all genes in the Brain cancer and Lymphoma data are not distinct in two classes, the different approaches considering different factors may select different discriminative gene subsets. Thus, the genes selected by the SC-IPSO-ELM are surely different from ones selected by other gene selected methods, which is verified by Tables 4 and 8.

**Fig. 2:**
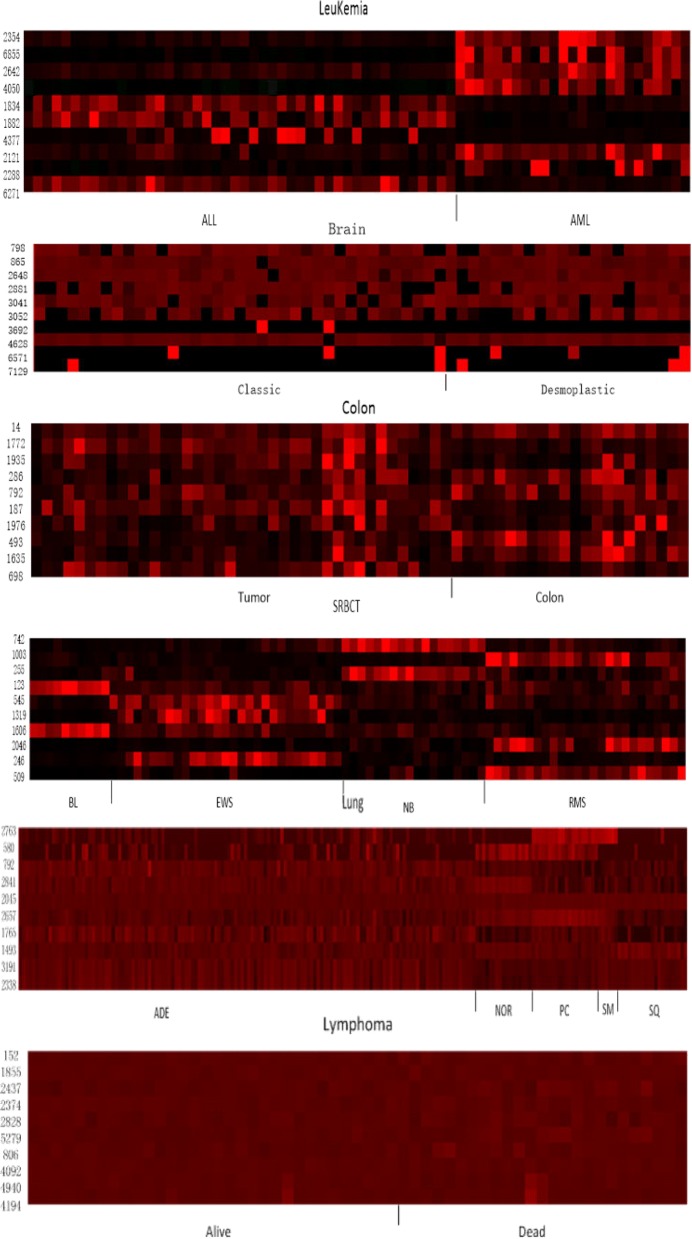
The heatmap of expression levels based on the top ten frequently selected genes on the six data

Figure 2 shows the heatmap with top ten frequently selected genes for the six data. It can be found that most of frequently selected genes’ expression levels clearly differentiate between/among two/multi classes on all data but the Brain cancer and Lymphoma data. From Fig. 2b and e, there has no single gene whose expression levels are distinct between two classes, which was verified in [15, 16]. Hence, the proposed method is capable of selecting predictive genes whose expression levels are distinct among different classes in most cases.

### Comparison with the GCSI based gene selection methods

In [15, 16], two effective gene selection methods by considering GCS information we proposed. Experimental results on several public microarray data verified that the two methods, the KMeans-GCSI-MBPSO-ELM and BPSO-GCSI-ELM methods, outperformed than some PSO-based methods and other classical gene selection methods such as GS2, GS1, Cho’s and F-test. To avoid repetition of the comparison with the PSO-based and other classical gene selection methods, the SC-IPSO-ELM method is compared with only the KMeans-GCSI-MBPSO-ELM and BPSO-GCSI-ELM methods on the six data by using ELM and support vector machine (SVM), and the corresponding results of the average of 100 trials are listed in Tables 9 and 10.

**Table 9:** The 5-fold cv classification accuracies of elm based on the three gene selection methods on the six microarray data

Data	KMeans-GCSI-MBPSO-ELM		BPSO-GCSI-ELM		SC-IPSO-ELM	
	5-fold CV Accuracy(%) ± std	genes	5-fold CV Accuracy(%) ± std	genes	5-fold CV Accuracy(%) ± std	genes
Leukemia	100.00 ±0.00	3	100.00 ±0.00	3	100.00 ±0.00	3
Brain cancer	88.63 ±0.0216	6	89.88 ±0.0223	7	91.88 ±0.019	7
Colon	97.61 ±0.0137	6	97.82 ±0.0132	9	99.05 ±0.011	13
SRBCT	100.00 ±0.00	6	100.00 ±0.00	6	100.00 ±0.00	6
LUNG	97.10 ±0.063	11	96.28 ±0.072	12	98.67 ±0.019	11
Lymphoma	86.97 ±0.024	8	84.50 ±0.023	8	93.79 ±0.020	7

**Table 10:** The classification accuracies of svm based on the three gene selection methods on the six microarray data

Data	KMeans-GCSI-MBPSO-ELM		BPSO-GCSI-ELM		SC-IPSO-ELM	
	5-fold CV Accuracy(%) ± std	genes	5-fold CV Accuracy(%) ± std	genes	5-fold CV Accuracy(%) ± std	genes
Leukemia	99.99 ±0.0014	3	99.99 ±0.0014	3	99.99 ±0.0014	3
Brain cancer	84.05 ±0.0301	6	82.70 ±0.0319	7	86.55 ±0.0299	7
Colon	90.69 ±0.0226	6	92.02 ±0.0275	9	93.35 ±0.0310	13
SRBCT	99.24 ±0.0119	6	98.34 ±0.0100	6	99.39 ±0.0074	6
LUNG	94.63 ±0.054	11	96.65 ±0.058	11	95.38 ±0.047	11
Lymphoma	77.59 ±0.032	8	72.41 ±0.034	8	81.03 ±0.025	7

From Tables 9 and 10, the SC-IPSO-ELM method selects the almost same number of genes as the two GCSI based methods on the Leukemia, Brain cancer, SRBCT, LUNG and Lymphoma data, while it selects the most number of genes on the Colon data among three methods. ELM achieves 100% 5-fold CV accuracy on the Leukemia and SRBCT data with the genes selected by the three methods, and SVM achieves the same 5-fold CV accuracy on the Leukemia data with the genes selected by the three methods. ELM and SVM both obtain the highest 5-fold CV accuracy on the Brain cancer, Colon data and Lymphoma data with the genes selected by the SC-IPSO-ELM method, SVM obtains the slightly higher 5-fold CV accuracy on the SRBCT data with the SC-IPSO-ELM than that with the two GCSI based methods, and SVM obtains the highest 5-fold CV accuracy on the LUNG data with the BPSO-GCSI-ELM. On the whole, the SC-IPSO-ELM could select more predictive gene subsets than the two GCSI based methods.

### Discussion on the parameter selection

**Fig. 3:**
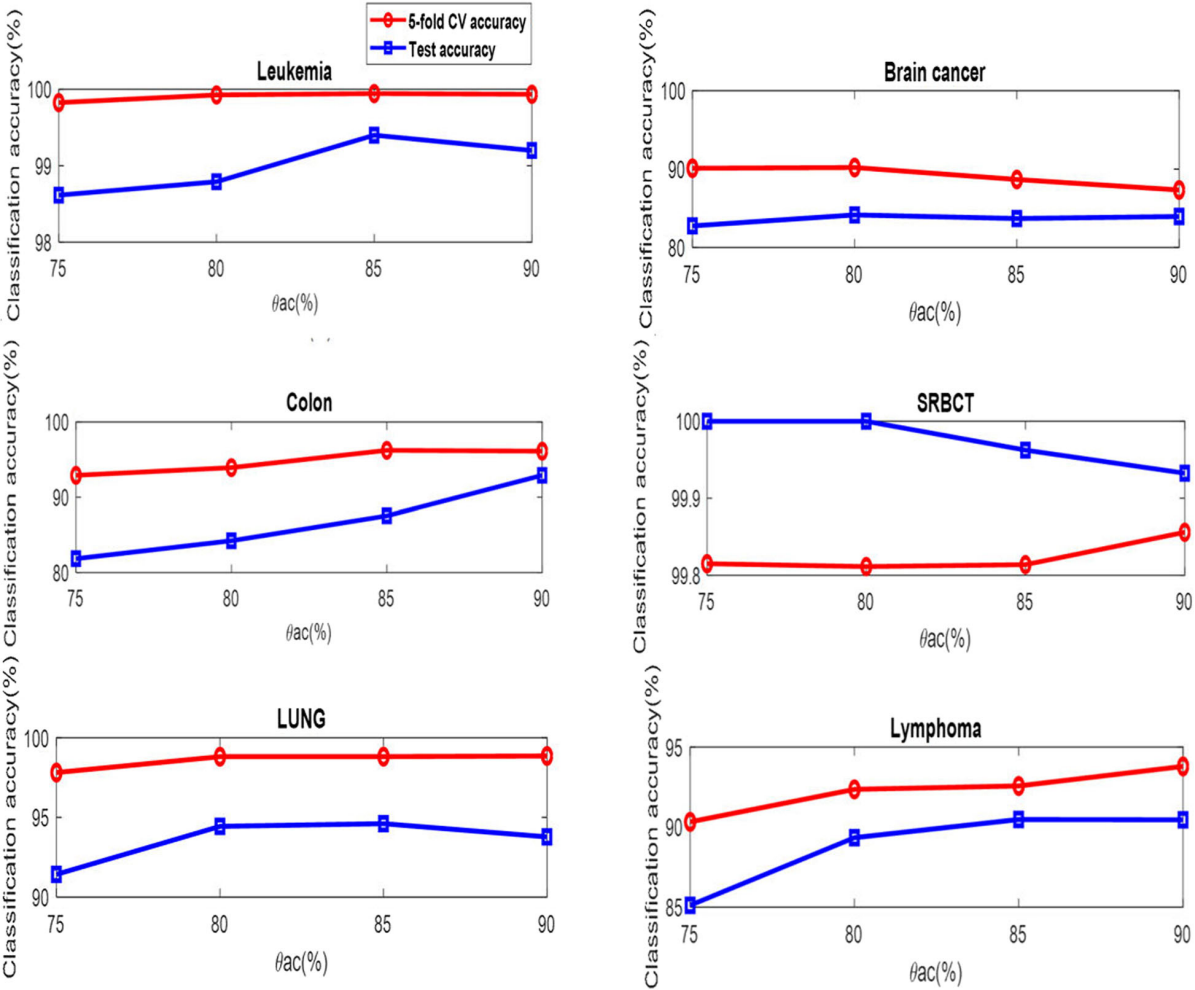
The parameter, *θ*ac versus the classification accuracy on the training dataset obtained by ELM

To establish second-level gene pool, it is critical to determine the value of the parameter, *θ*_*ac*_. Figure 3 shows the relationship between the classification accuracy on the training data obtained by ELM and the parameter, *θ*_*ac*_. On the Leukemia, Colon data, LUNG and Lymphoma data, the 5-fold CV and test accuracy both have an upward trend as the values of the parameter, increases, while they have a downward trend as the values of the parameter increases on the Brain cancer data. On the SRBCT data, the test accuracy decreases as the value of the parameter increases, while the 5-fold CV accuracy increases as the value of the parameter increases.

**Fig. 4:**
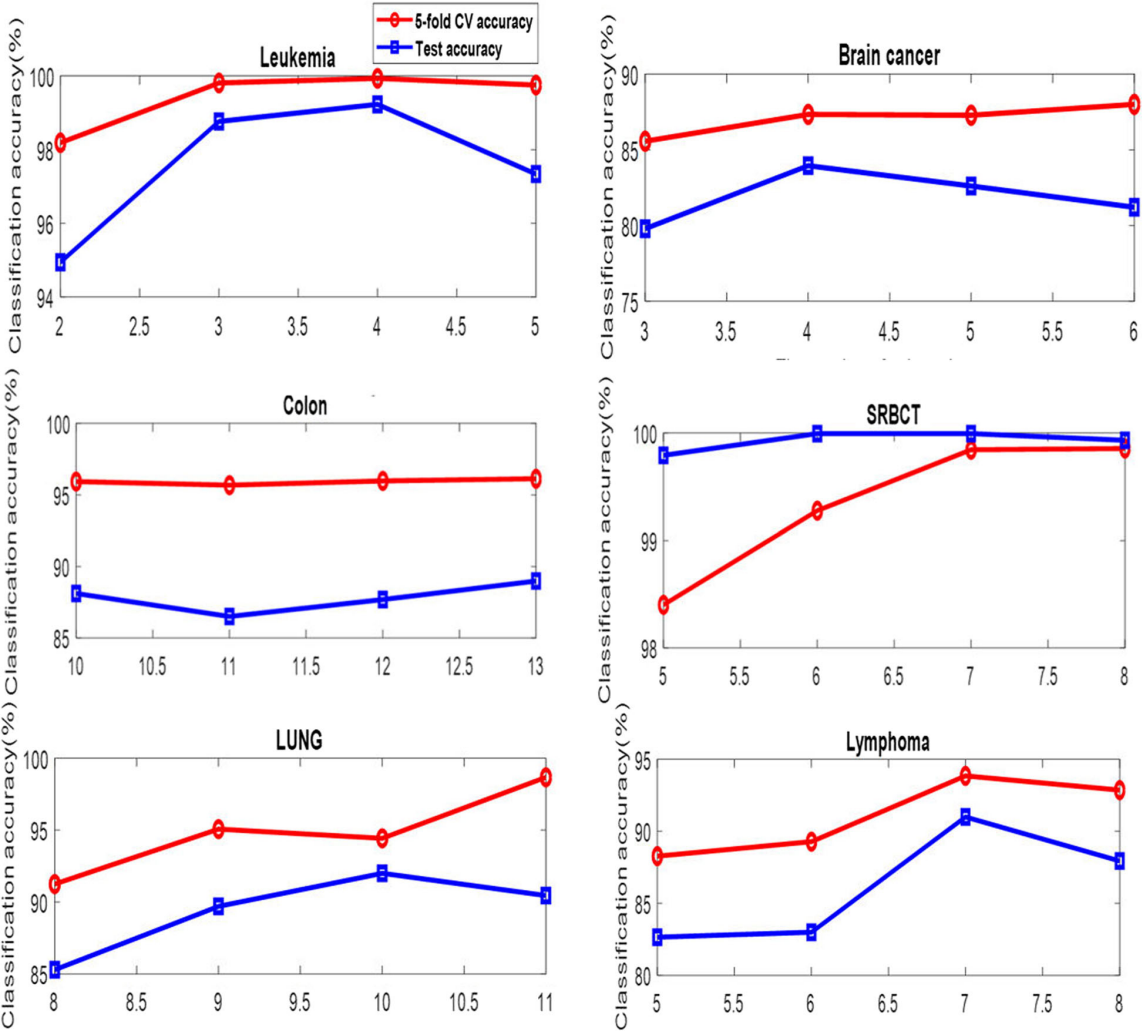
The number of the selected genes versus the classification accuracy on the training dataset obtained by ELM

For using the improved PSO to select the gene subset, the dimension of the particle is the number of the selected genes. Figure 4 shows the effect on different number of the selected genes. The 5-fold CV accuracy obtained by ELM has an upward trend as the number of the selected genes increases on the six data but the Colon data, while the curves of the test accuracy obtained by ELM fluctuate as the number of the selected genes increases on the six data.

Figures 3 and 4 provide a guide on how to select the values of the parameters *θ*_*ac*_ and the number of the selected genes in the SC-IPSO-ELM. In general, these parameters should be selected empirically in particular applications.

## Conclusions

To obtain predictive genes with lower redundancy, a hybrid gene selection method based on gene scoring strategy and improved PSO was proposed in this paper. To decrease the computational cost, the relevant genes are filtered out through different strategies to establish more compact gene pool for further gene selection. Then, the improved PSO was proposed to select the most predictive gene subsets from the gene pool. Experimental results verified the proposed method could select highly predictive and compact gene subsets and outperformed than other PSO-based and GCSI-based gene selection methods. However, the proposed method selects genes lack of much interpretability. Future work will include how to encode some prior information into the proposed method for gene selection and apply it to RNA-Seq data analysis.
